# The Work of London and Provincial Hospitals

**Published:** 1914-10-10

**Authors:** 


### HOSPITALS AND THE WAR.

(From our Special Correspondents.)

### The Work of London and Provincial Hospitals.

### HAMPSTEAD GENERAL HOSPITAL.

The hospital has received twenty wounded soldiers from the Royal Herbert Hospital, Woolwich. These patients were conveyed to the hospital under the care of Dr. C. J. R. MacFadden, Commandant V.A.D. London 11, assisted by members of the St. John Ambulance Brigade, No. 1 District. They were transported in the hospital's own motor-ambulance and twelve motor-cars, which had been kindly lent by residents in the district.

The wounded soldiers were received and welcomed by the Grand Duke Michael, the president of the Hampstead General Hospital, and they are now comfortably settled in the wards which have been specially set apart for their accommodation. The hospital still continues to receive responses to the appeal made by the Grand Duke for additional funds to maintain the work, but further help is urgently needed. The cost of maintenance has been, of course, increased owing to the rise in the price of provisions and other necessaries consequent on the war.

### THE WOMEN'S WAR HOSPITAL.

A hospital unit, sent out by the Women's National Service League, has left England for Antwerp, which is entirely staffed by women. When other somewhat similar units have gone to the Continent to help the wounded they have been accompanied by men doctors. To be strictly accurate, however, we are also informed that among the company of women leaving England there were two men, the electricians responsible for the working of the *x*-ray apparatus. Mrs. St. Clair Stobart is in charge of the party. The unit is composed of twenty-eight women. There are six doctors and surgeons: Dr. Florence Stoney, M.D., B.S., an *x*-ray operator; Dr. Hanson, head surgeon; Dr. Rose Turner, L.R.C.P. and S.; Dr. Emily Morris, M.D., B.S.; Dr. Ramsay, and Dr. Joan Watts. In addition to the doctors are twelve qualified nurses and ten orderlies.

The hospital equipment included an *x*-ray apparatus and 130 beds, besides boxes of drugs, stores, and clothing. The British Minister at Antwerp, Sir Cecil Hertslet, is stated to have invited Mrs. St. Clair Stobart to bring her hospital unit to the aid of the hardly pressed Belgian Red Cross, to which, from the great numbers of wounded arriving at Antwerp, it ought to be of much service.

The headquarters of the Women's National Service League, who, as stated, are sending out the expedition, are at 39 St. James's Street.

